# Hereditary Xerocytosis: Differential Behavior of PIEZO1 Mutations in the N-Terminal Extracellular Domain Between Red Blood Cells and HEK Cells

**DOI:** 10.3389/fphys.2021.736585

**Published:** 2021-10-18

**Authors:** Yohei Yamaguchi, Benoit Allegrini, Raphaël Rapetti-Mauss, Véronique Picard, Loïc Garçon, Peter Kohl, Olivier Soriani, Rémi Peyronnet, Hélène Guizouarn

**Affiliations:** ^1^Institute for Experimental Cardiovascular Medicine, University Heart Center Freiburg, Bad Krozingen, Germany; ^2^Medical Center and Faculty of Medicine, University of Freiburg, Freiburg im Breisgau, Germany; ^3^Université Côte d’Azur, CNRS, INSERM, Institut de Biologie Valrose, Nice, France; ^4^Université Paris Sud-Paris Saclay, Faculté de Pharmacie, Service d’Hématologie Biologique, Hôpital Bicêtre, APHP, Le Kremlin-Bicêtre, France; ^5^Université Picardie Jules Verne, EA 4666, Service d’Hématologie Biologique, CHU, Amiens, France; ^6^CIBSS – Centre for Integrative Biological Signalling Studies, University of Freiburg, Freiburg im Breisgau, Germany

**Keywords:** PIEZO1, KCNN4, hereditary xerocytosis, stomatocytosis, red blood cell

## Abstract

Hereditary Xerocytosis, a rare hemolytic anemia, is due to gain of function mutations in PIEZO1, a non-selective cation channel activated by mechanical stress. How these PIEZO1 mutations impair channel function and alter red blood cell (RBC) physiology, is not completely understood. Here, we report the characterization of mutations in the N-terminal part of the protein (V598M, F681S and the double mutation G782S/R808Q), a part of the channel that was subject of many investigations to decipher its role in channel gating. Our data show that the electrophysiological features of these PIEZO1 mutants expressed in HEK293T cells are different from previously characterized PIEZO1 mutations that are located in the pore or at the C-terminal extracellular domain of the protein. Although RBC with PIEZO1 mutations showed a dehydrated phenotype, the activity of V598M, F681S or R808Q in response to stretch was not significantly different from the WT channels. In contrast, the G782S mutant showed larger currents compared to the WT PIEZO1. Interestingly, basal activity of all the mutated channels was not significantly altered at the opposite of what was expected according to the decreased water and cation contents of resting RBC. In addition, the features of mutant PIEZO1 expressed in HEK293 cells do not always correlate with the observation in RBC where PIEZO1 mutations induced a cation leak associated with an increased conductance. Our work emphasizes the role of the membrane environment in PIEZO1 activity and the need to characterize RBC permeability to assess pathogenicity to PIEZO1 mutants associated with erythrocyte diseases.

## Introduction

Gain-of-function mutations in PIEZO1 have been linked to Dehydrated Hereditary Stomatocytosis (DHSt), a rare red blood cell (RBC) disease also known as hereditary xerocytosis (Zarychanski et al., 2012; Albuisson et al., 2013; Andolfo et al., 2013). PIEZO1 is a non-selective cation channel, activated by mechanical stimuli, that has been proposed to link mechanical forces to RBC calcium permeability (Coste et al., 2012; Cahalan et al., 2015). Once activated, PIEZO1 mediates a cation current that decreases toward basal values even during persistent mechanical stimulation (inactivation). In RBC, this transient activation of PIEZO1 in response to membrane stretch increases intracellular calcium concentration and stimulates the Ca^2+^ activated K^+^ channel, KCNN4 (or Gardos channel), that allows K^+^ to leave the cell. The high anion conductance of RBC membrane allows Cl^–^ flux, leading to a net KCl efflux. This loss of osmolytes from the cytosol is accompanied by water leaving the cell, resulting in RBC dehydration. Gain-of-function mutations in PIEZO1 have thus been associated with RBC dehydration through excessive KCNN4 stimulation (Rapetti-Mauss et al., 2017).

In DHSt patients with PIEZO1 gain of function mutations, RBC are dehydrated and show a typical leftward shift in osmotic gradient ektacytometry (i.e., toward increased deformability at lower osmolality values), attesting changes in the water content equilibrium (Andolfo et al., 2013; Picard et al., 2019; Pérès et al., 2021). The increasing use of sequencing in diagnosis has resulted in identification of a collection of mutations in *PIEZO1*. Characterizing the electrophysiological features of new PIEZO1 mutations, discovered by genetic screening of patients with suspected DHSt, is necessary to understand the link between the genotype and the RBC phenotype.

Here, we report electrophysiological features of five different PIEZO1 mutations, identified in three independent index cases with typical DHSt clinical and biological phenotypes [as described in previous publications (Andolfo et al., 2013; Rapetti-Mauss et al., 2017)]. In contrast to the majority of PIEZO1 mutations associated to DHSt so far, the present mutations are located at the N-terminal part of the protein. This part of the protein is not yet structurally resolved, however, it is made up of clusters of alpha helices, that may function as membrane tension sensors (Zhao et al., 2018). Recent structural studies proposed that this domain is organized as a repetitive pattern of 4 helices connected by intracellular and extracellular loops similar to the 6 repeats of 4 helices that shape the extended arm of each subunit of the channel (Guo and MacKinnon, 2017). Interestingly, the electrophysiological features of here assessed PIEZO1 mutants (expressed in HEK293 cells) are different from previously characterized PIEZO1 mutations that are located in the pore or at the C-terminal extracellular domain of the protein.

## Materials and Methods

Blood samples: Human blood samples were obtained with written informed consent from four hereditary xerocytosis patients from previously described families (Andolfo et al., 2013) and six unrelated healthy control subjects. Blood withdrawn into EDTA collecting tubes was used directly for ektacytometry in a hospital laboratory and sent to the research laboratory within 24 h. Osmotic gradient ektacytometry was performed on fresh blood samples as previously described (Cynober et al., 1996). In brief, red blood cells in a 4% polyvinylpyrrolidone solution of gradually increasing osmolality (from 60 to 450 mOsm) were submitted to continuous flow using a first generation ektacytometer (Bayer Diagnostics, Tarrytown, NY, United States) and osmolar parameters as well as maximal elongation index were recorded. Upon reception at the research lab, blood was washed three times in saline solution containing (in mM): NaCl (145), KCl (5), MgSO_4_ (2), CaCl_2_ (1), HEPES (10) pH 7.4 and any buffy coat was removed by aspiration. RBC Na^+^, K^+^ and water content were measured as previously described (Rapetti-Mauss et al., 2015).

HEK293T cell experiments: HEK293T cells were transiently transfected with pIRES-eGFP plasmids containing the following PIEZO1 constructs: the control PIEZO1 channel WT, V598M, F681S, G782S, R808Q, or G782S/R808Q. Transfection was conducted using jetPEI transfection reagent (Polyplus-transfection) and experiments were carried on 48 to 72 h after transfection. pIRES-eGFP containing human WT PIEZO1 DNA was a kind gift from B. Coste. Mutations in the WT PIEZO1 were introduced by PCR with pfu Turbo DNA polymerase and primers overlapping the mutation by 15 nucleotides downstream and upstream. The obtained constructs were sequenced to check for correct mutations (Beckman Coulter Genomics, Takeley, United Kingdom).

To quantify PIEZO1 expression at the plasma membrane, HEK293T cells transfected with the different plasmids or not transfected (NT) were labeled with biotin using Pierce cell surface protein isolation kit according to manufacturer’s procedures (Pierce Thermo Fisher). 48 h after transfection, cells at 80% confluence were washed twice with ice-cold Phosphate Buffered Saline (PBS) and incubated for 30 min on ice with sulfo-NHS-SS-biotin in PBS. Labeled proteins were immunodetected by Western blot, using anti-PIEZO1 antibody 1/1,000 (Proteintech 15939-1-AP) and anti Na^+^/K^+^ ATPase ß1 subunit 1/1,000 (Sigma Aldrich) was used as an internal loading standard. The intensity of biotinylated Na^+^/K^+^ ATPase (I_NaK_) and PIEZO1 (I_P_) lanes were quantified using Fiji software (Schindelin et al., 2012). A normalizing factor (Nf = I_NaK_ in lanes with WT PIEZO1/I_NaK_ in lanes with mutant PIEZO1) was calculated to normalize the PIEZO1 signal. The ratio (I_p mutant_ × Nf)/I_p WT_ was calculated and plotted for five different Western blots corresponding to three different preparations of HEK293T cell lysates.

Electrophysiology: stretch-activated currents of PIEZO1 WT and mutants in HEKT293 cells, 48–72 h after transfection, were recorded in cell-attached configuration. Current recordings were done using an Axopatch 200B Microelectrode Amplifier (Axon Instruments). Currents were induced by stepwise negative pipette pressure (0 to −80 mmHg, Δ10 mmHg, 500 ms), applied using a high-speed pressure clamp (ALA Scientific Instruments). Average pipette resistance was 1.3 ± 0.3 MΩ. Peak current-pressure relationship of stretch-activated currents elicited at −80 mV were fitted with a Boltzmann equation. Currents were acquired at 20 kHz sampling rate, and low-pass filtered at 1 kHz. Recordings were analyzed with pCLAMP 10.6 software (Axon Instruments) using solutions already described for characterizing PIEZO1 channels (Peyronnet et al., 2013). The bath solution contained (in mM): KCl (155), MgCl_2_ (3), EGTA (5), HEPES (10); pH buffered to 7.2 using KOH, measured osmolality ≈ 320 mOsm L^–1^. The solution was stored at room temperature. Pipette solution, contained (in mM): NaCl (150), KCl (5), HEPES (10), CaCl_2_ (2), pH buffered to 7.4 using NaOH, ≈ 305 mOsm L^–1^.

## Results

**FIGURE 1:**
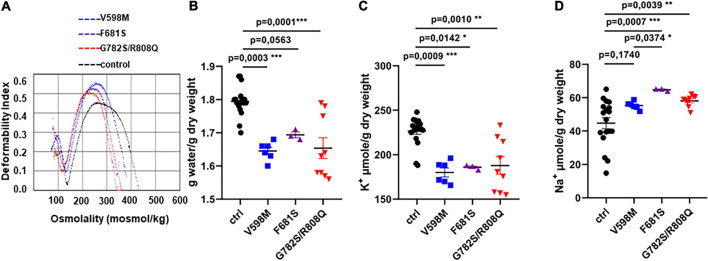
**(A)** Ektacytometry profiles of RBC from three independent index-cases with clinical and biological phenotypes typical of hereditary xerocytosis, which were previously described. **(B)** water, **(C)** K^+^, and **(D)** Na^+^ content of control or mutant RBC, measured in resting conditions 24 h after blood withdrawal. Statistical analyzes of the data were done with a Kruskal and Wallis test followed by a Dunn’s post-test. The *p* value is given on each figure. There were no significant differences (*p* < 0.05) between mutants except the Na^+^ content between V598M and F681S, *p* = 0.03.

We first analyzed RBC from three patients who carried the following PIEZO1 substitutions: V598M, F681S and the double mutation G782S/R808Q, respectively. Patients’ RBC ektacytometry curves were typical of DHSt (Figure 1A), showing a leftward shift of the maximum elongation index indicative of cell dehydration (already shown for V598M and F681S in Rapetti-Mauss et al., 2017). Indeed, patients’ RBC water content was about 10% lower for V598M and G782S/R808Q mutations, compared to WT RBC (Figure 1B). A significantly lower K^+^ content was observed for all three mutants, with a high variability for G782S/R808Q (Figure 1C). Na^+^ content was significantly higher (∼10–15%) in the F681S and the double mutant (Figure 1D). When observed, this Na^+^ increase did not fully compensate the K^+^ loss, and there is a decrease in monovalent cation content (Na^+^ + K^+^), which correlates with the RBC dehydration. It was shown in our previous work, that patients’ RBC cation leak is associated with a rise in cell membrane conductance (Rapetti-Mauss et al., 2017): a transient current following Goldman-Hodgkin-Katz law was observed in patients’ RBC, which is not observed in control RBC (recorded in whole cell configuration). This current is thought to be indicative of increased PIEZO1 activity in RBC with the PIEZO1 mutations V598M, F681S and G782S/R808Q. The increased current may result either from a higher stretch sensitivity, a greater conductance of mutant PIEZO1, an increased presence of channels in the cell membrane, or a mix of these factors. Thus far, the stretch-sensitivity of the PIEZO1-mutants has not been characterized.

To analyze the functional consequences of these PIEZO1 mutations, HEK293 cells were transiently transfected with pIRES-eGFP vector containing either the control human PIEZO1 (WT), the mutants V598M, F681S, G782S/R808Q as in RBC or G782S and R808Q alone.

**FIGURE 2:**
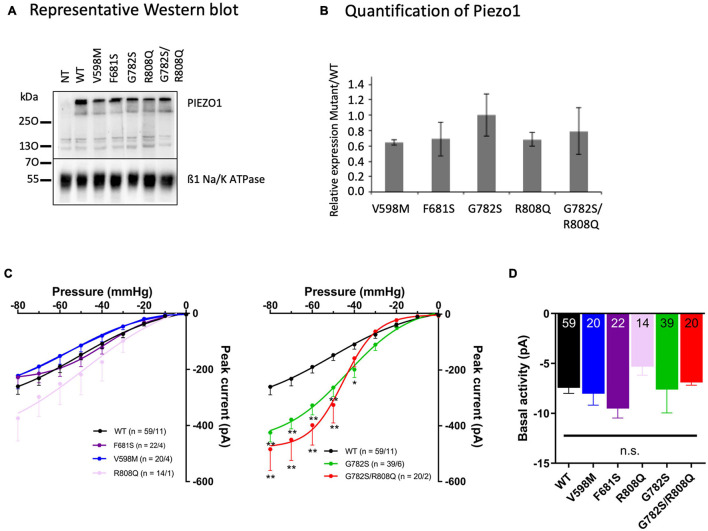
**(A)** Representative Western blot (WB) from three different cell labeling done on three different batches of transfected cells. **(B)** Quantification of PIEZO1 mutant expression related to WT. A ratio of Na^+^/K^+^ ATPase signal for each mutant versus WT was calculated for each WB and the PIEZO1 signal was corrected according to this internal standard. The plot illustrates the ratio Mutant/WT PIEZO1 calculated after five different WB (means ± sem). Statistical analyzes were done with Kruskal and Wallis test and differences were not significant (*p* > 0.05). **(C)** Stretch-activated currents of PIEZO1 WT and mutants, expressed in HEK293T cells, recorded in cell-attached configuration. Bars represent the mean ± sem, and the number of cells tested/the number of cell transfections is shown as n in the figures. **p < 0.01; *p < 0.05 vs. WT, two-way analysis of variance with Bonferroni’s *post hoc*. **(D)** Basal activity of PIEZO1. Basal activity calculated from the current traces without additional negative pipette pressure application, showing no significant differences between eGFP without PIEZO1, WT, and mutant constructs. Bars represent the mean ± sem, and the number of cells tested is shown in the bottom of the bars. n.s., not significantly different, one-way analysis of variance with Bonferroni’s *post hoc*.

The expression and location at the plasma membrane of WT and mutant PIEZO1 protein were assessed by quantification of biotinylated PIEZO1, and related to the expression of the ß1 subunit of Na^+^/K^+^ ATPase. There was no significant difference between WT and mutant PIEZO1 levels (Figures 2A,B) in protein expression at the plasma membrane. A representative WB of total and biotinylated PIEZO1 signal with a quantification of PIEZO1 signal is shown in Supplementary Figures 1A,B. An immunofluorescence labeling of PIEZO1 in HEK293 transfected cells illustrated the transfection efficiency (Supplementary Figure 2). Thus, mutant protein trafficking and targeting to the HEK293 plasma membrane was similar to WT. Of note, we did not detect endogenous PIEZO1 protein by Western blot in our HEK293 cell line.

**FIGURE 3:**
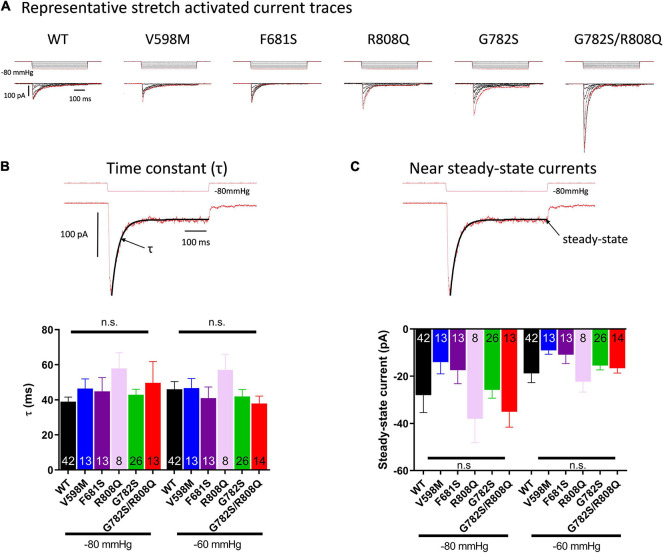
**(A)** Representative traces of currents induced by stepwise negative pipette pressure (0 to −80 mmHg, Δ10 mmHg) recorded in HEK293T cells expressing WT and mutants. Stretch-activated currents of PIEZO1 WT and mutants-expressing HEK293T cells recorded in cell-attached configuration. **(B)** Inactivation time constant (τ) of currents from PIEZO1 WT and mutants. The top trace shows a representative current fitted by an exponential decay at −60 and −80 mmHg. τ is obtained from the fit. **(C)** Near steady-state currents from PIEZO1 WT and mutants. The steady-state current values were obtained from the fit at the end of the pressure pulse. Currents recorded in cell-attached configuration.

Stretch-activated currents of each PIEZO1 mutant were measured using stepwise application of negative pressure in cell-attached configuration. The peak current in V598M, F681S and R808Q was not significantly different from WT (Figure 2C), while in G782S and G782S/R808Q it was significantly larger at pipette pressures ranging from −40 to −80 mmHg. Representative current traces for each construct are shown in Figure 3A. In the absence of negative pipette pressure, the current levels of WT and mutant PIEZO1 channels were not significantly different (Figure 2D), suggesting no change in constitutive current activity of mutated channels in HEK293 cells. The inactivation kinetics of channels, as well as their near steady-state currents, were not statistically different in WT and mutants (Figures 3B,C).

## Discussion

Data presented here show that the Na^+^ and K^+^ permeability of patients’ RBC was increased in the three cases of PIEZO1 mutations (V598M, F681S, G782S/R808Q) and these RBC are slightly dehydrated due to chronic activation of KCNN4, as shown in our previous publication (Rapetti-Mauss et al., 2017). The properties of these RBC are similar to the ones recently described for the D669Y PIEZO1 mutant (Pérès et al., 2021). The D669Y PIEZO1 RBC have an increased cation conductance and slight dehydration likely due to more frequent activation of KCNN4. Hence, these PIEZO1 mutants are likely to induce a constitutive Na^+^, K^+^, and Ca^2+^ leak in RBC. Moreover, in previously published patch-clamp experiments on RBC carrying the V598M PIEZO1 mutant (in whole cell and cell-attached configurations), a constitutive cation leak, increased conductance and increased spontaneous activity were observed (Rapetti-Mauss et al., 2017; Gnanasambandam et al., 2018). To characterize mutant PIEZO1 features, they were expressed in HEK293 cells.

Once expressed in HEK293 cells, peak current and kinetics in response to stretch of PIEZO1 mutants V598M, F681S, and R808Q did not differ significantly from WT. Only the PIEZO1 constructs containing G782S either alone or as double mutation G782S/R808Q showed an increased peak current but no significant change in inactivation kinetics compared to the WT (Figure 3). In the presence of the double mutation G782S/R808Q only the G782S mutation changed PIEZO1 response to stretch, suggesting a negligible effect of the R808Q substitution in this response. Translated to RBC, only the G782S mutation showing an increased peak current, would be expected to rise intracellular Ca^2+^ and Na^2+^, and lose K^+^, as PIEZO1 is a cation non-selective channel. The increase in Ca^2+^ could induce an additional K^+^ leak through KCNN4 activation and lead to the observed RBC dehydration.

These data show that there are different responses in RBC and expression systems. Such cell type-dependence of the behavior of PIEZO1 mutants may be explained by differences in cell shape, lipid composition, and surface tension of RBC and HEK293 cells, as the position of the mutated amino acids in PIEZO1 suggests a role for these residues in membrane tension sensing, which may be affected by membrane composition and lipid or cytoskeleton interactions. In HEK293 cells, the cytoskeleton is indeed altering PIEZO1 gating (Cox et al., 2016). Lipid bilayer forces drive conformational changes in PIEZO1, and cholesterol-PIEZO1 interactions have been proposed to control the spatiotemporal activity of the ion channel (Cox et al., 2016; Buyan et al., 2020; Ridone et al., 2020). Moreover fatty acid composition of the bilayer also modulates PIEZO1 mechanical response (Romero et al., 2019). The different status of PIEZO1 in HEK293 cells and RBC is confirmed further by the effect of Yoda1, a PIEZO1 activator, which activates the channel in resting RBC and other cell types (Li et al., 2014; Rapetti-Mauss et al., 2017; Blythe et al., 2019; Atcha et al., 2021), whereas in HEK293 cells Yoda1-effects on PIEZO1 activation are observed only in the presence of additional membrane stretch (Syeda et al., 2015; Botello-Smith et al., 2019). Although HEK293 cells constitute a powerful expression system extensively used to study stretch-activated channels (and channels in general), these above considerations together with the results presented here question the relevance of using HEK293 cells to study the gating of mechano-sensitive channel from RBC. Of note, in a previous study, the pressure sensitivity of V598M PIEZO1 mutant expressed in HEK293 cells was shown to slightly increase compared to WT (Gnanasambandam et al., 2018). This discrepancy with our present results would suggest an even more complex regulation of PIEZO1 activity depending on the different experimental conditions among diverse laboratories, such as (i) different cellular clones, (ii) stages of cellular cycle, (iii) cellular senescence, and (iv) number of replicates.

The mutations are in the more distal part of the arms of the channel where it connects with the membrane curvature. According to the dome mechanism (Lin et al., 2019), changes in this domain might alter tension sensitivity of the channel (Haselwandter and MacKinnon, 2018). The specificity of RBC cytoskeleton (Svetina, 2020) and PIEZO1 interaction with spectrin (Dumitru et al., 2021) are likely responsible for differential sensitivity of the arms of the channel compared to HEK293 cells. The PIEZO1/spectrin relation is emphasized by the recent study showing a coinheritance of PIEZO1 and spectrin mutations in a cohort of 155 patients with clinical suspicion of hereditary anemia (Andolfo et al., 2021).

Although electrophysiological features of PIEZO1 mutants expressed in HEK293 cells may differ from RBC, expression systems remain helpful for studying PIEZO1. Differences in PIEZO1 electrophysiological features between the two cell types might furthermore point out residues involved in channel regulation by its environment.

Our data suggest that a direct analysis of patients’ RBC electrophysiological features is required to assess specifically the RBC pathogenicity of PIEZO1 mutations. The use of automated patch-clamp devices to test PIEZO1 in RBC may be a promising strategy to analyze the increasing number of PIEZO1 mutations identified in patients with suspected DHSt (Rotordam et al., 2018).

## Data Availability Statement

The original contributions presented in the study are included in the article/Supplementary Material, further inquiries can be directed to the corresponding author.

## Author Contributions

YY and RP performed patch clamp experiments on HEK293T cells. BA, RR-M, OS, and HG performed experiments on red blood cells. VP and LG took care of patients and provided blood samples. VP did the ektacytometry. PK, RP, RR-M, and OS supervised electrophysiology studies. HG supervised the study and wrote the manuscript. All authors analyzed data and improved the manuscript.

## Conflict of Interest

The authors declare that the research was conducted in the absence of any commercial or financial relationships that could be construed as a potential conflict of interest.

## Publisher’s Note

All claims expressed in this article are solely those of the authors and do not necessarily represent those of their affiliated organizations, or those of the publisher, the editors and the reviewers. Any product that may be evaluated in this article, or claim that may be made by its manufacturer, is not guaranteed or endorsed by the publisher.
