# Supplementary figures and images for: Hereditary Xerocytosis: Differential Behavior of PIEZO1 Mutations in the N-Terminal Extracellular Domain Between Red Blood Cells and HEK Cells
Source: Front Physiol. 2021 Oct 18;12:736585. doi: 10.3389/fphys.2021.736585 (PMC8562563; doi:10.3389/fphys.2021.736585)

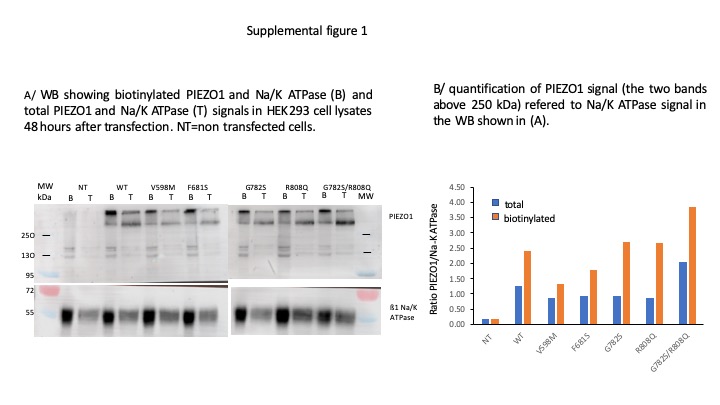

Supplement: Supplementary file 1 [file Image_1.JPEG]

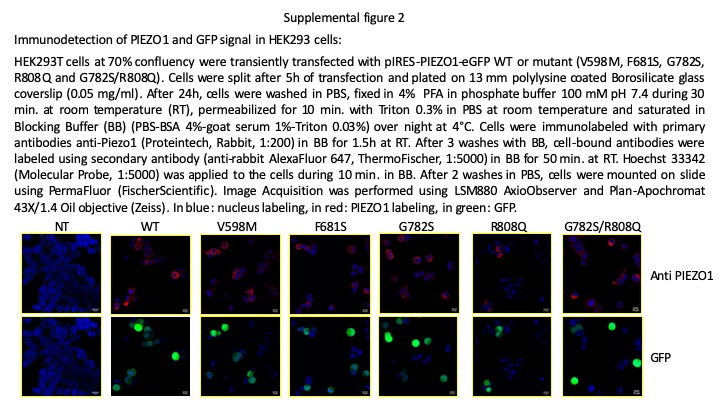

Supplement: Supplementary file 2 [file Image_2.JPEG]
